# A High-Precision Method for 100-Day-Old Classification of Chickens in Edge Computing Scenarios Based on Federated Computing

**DOI:** 10.3390/ani12243450

**Published:** 2022-12-07

**Authors:** Yikang Huang, Xinze Yang, Jiangyi Guo, Jia Cheng, Hao Qu, Jie Ma, Lin Li

**Affiliations:** 1 College of Information and Electrical Engineering, China Agricultural University, Beijing 100083, China; 2 College of Economics and Management, China Agricultural University, Beijing 100083, China; 3 College of Agronomy and Biotechnology, China Agricultural University, Beijing 100083, China; 4 Institute of Animal Science, Guangdong Academy of Agricultural Sciences, Guangzhou 510640, China

**Keywords:** 100-day-old classification of chickens, edge computing scenarios, federated learning, poultry, deep learning

## Abstract

**Simple Summary:**

Improving the accuracy of day-age detection of chickens is of great importance for chicken rearing. This paper focuses on the problem of classifying the age of chickens within 100 days. This paper proposes a high-precision federated learning-based model that can be applied to edge computing scenarios. Finally, our method can achieve an accuracy of 96.1%, which can fully meet the needs of application scenarios.

**Abstract:**

Due to the booming development of computer vision technology and artificial intelligence algorithms, it has become more feasible to implement artificial rearing of animals in real production scenarios. Improving the accuracy of day-age detection of chickens is one of the examples and is of great importance for chicken rearing. This paper focuses on the problem of classifying the age of chickens within 100 days. Due to the huge amount of data and the different computing power of different devices in practical application scenarios, it is important to maximize the computing power of edge computing devices without sacrificing accuracy. This paper proposes a high-precision federated learning-based model that can be applied to edge computing scenarios. In order to accommodate different computing power in different scenarios, this paper proposes a dual-ended adaptive federated learning framework; in order to adapt to low computing power scenarios, this paper performs lightweighting operations on the mainstream model; and in order to verify the effectiveness of the model, this paper conducts a number of targeted experiments. Compared with AlexNet, VGG, ResNet and GoogLeNet, this model improves the classification accuracy to 96.1%, which is 14.4% better than the baseline model and improves the Recall and Precision by 14.8% and 14.2%, respectively. In addition, by lightening the network, our methods reduce the inference latency and transmission latency by 24.4 ms and 10.5 ms, respectively. Finally, this model is deployed in a real-world application and an application is developed based on the wechat SDK.

## 1. Introduction

The modern chicken breeding industry presents the characteristics of high intensification and integration. With the requirements and challenges of the food crisis, environmental protection, biosecurity and animal welfare, the modern chicken breeding industry urgently needs to be transformed from labor-intensive to intelligent [1].

The day-age of a chicken is a concept similar to human age [2]. It is an important indicator of its growth. The day-age of a chicken plays an essential role in modern chicken farming, which is related to feed conversion, reproduction traits and slaughter performance. These indexes also directly affect the production management of chickens and achieve optimal poultry production. Therefore, many chicken breeding enterprises will regularly recognize the day-age of chickens and conduct group management in order to realize precision feeding, which can help enterprises to reduce costs and increase efficiency.

Generally, chickens can be divided into laying hens and broilers by their production specialists. Broiler production mainly adopts the system named “all in, all out” without involving herd transfer. Laying hens [3] can be divided into reserve chickens which are from newborn to 126 day age and laying hens which are from 127 to 504 day age. Reserve chicken is a crucial essential stage in laying hen production. Different types of laying hens not only directly affect the growth and development of laying hens, but also affect the production performance during the laying period. For laying hens, it also affects the breeding value, the renewal of the flock and the smooth completion of the production plan. According to the different environmental conditions and nutritional needs of breeding, detailed classification information of reserve chickens is displayed in Table 1.

By dividing chickens into different stages according to their day-age, this paper carries out targeted feeding management, which is of great significance to improve feeding efficiency. These significance is mainly reflected in the following aspects:

1. Determine the utilization cycle of breeding chickens [4], the feeding cycle of laying hens and the best slaughter time of broilers [1].
2. Improve the efficiency of feed utilization [5] and reduce the feed cost and the environmental pollution caused by chicken excreta.
3. Predict possible diseases at each stage [6,7] and administer the targeted prevention programs.
4. Provide precision feeding for the differences in light [8], temperature and drinking water needs at each stage.

In traditional chicken farming, the day-age of chickens is usually estimated by the experience of the poultry feeders. The most intuitive index is the weight of chickens. Under the same rearing conditions, the weight of chickens of the same sex at a certain day-age is within a certain range. Some physical features can also be used to judge the day-age. Here are four traditional methods of determining the day-age of a chicken:

1. Beak. In free-ranging flocks, the beak of the short-day-age chickens is taper, narrow and thin and there is no hard horn; As a result of long-term outdoor foraging, the beak of the long-day-age chicken is thick and short, the end becomes hard and smooth and the two sides are broad and rough.
2. Crest. The crests of chickens with a short day-age are smaller, while the crests grow larger with the increase in day-age.
3. The length of the feather. The feather length of chickens will elongate with the increase in the day-age of chickens and the day-age of chickens can be roughly judged by the main wing feathers [9].
4. Metatarsus. The metatarsal length is positively correlated with chicken day-age and elongates with the increase in chicken day-age. Metatarsal scales are soft when young and keratinized when adults. The larger the day-age, the harder the scales and they even protrude laterally.

However, these methods are derived from experience, which may have large personal errors and poor accuracy. They can only be used as rough judgment and cannot accurately determine the day-age of chickens, so they are not suitable for the needs of precision feeding in the chicken breeding industry.

In large-scale farms, physical barriers are often used to separate flocks of chickens of different day-ages, strict production records are made to ensure accurate monitoring of the day-age of chickens and regular inspections are carried out to eliminate or transfer the chickens that do not conform to the uniformity of the flock. However, this is undoubtedly labor-intensive and will bring about a series of animal welfare problems [10]. With the development of computer vision and its application in the chicken breeding industry, the optical density assay method has been used to measure the emission density of bone [11] to infer the day-age of chickens. However, this method of measuring bone density cannot be applied to live chickens.

Based on this, modern chicken breeding needs a method that can get rid of the empirical aspect and accurately identify the day-age of chickens. Therefore, utilizing artificial intelligence to establish a set of less personal errors and a human contact chicken day-age identification method has positive significance for modern chicken farming [12]. There are great differences in the feeding and management methods of laying hens at different day-ages [13]. During the reserve chicken period, the requirement of different amino acids is different in different periods, which needs to be accurately grasped [14]. Thirdly, the accurate identification of chicken day-age is helpful to accurately determine the broiler production time and the elimination time of laying hens, which can improve the economic benefits. The meat quality of broilers varies depending on the different day-age at which they are slaughtered, divided and sold [15]. By determining the slaughter time through the accurate day-age judgment, the texture needs of different chicken products can be grasped dynamically in the market, so that they can provide chickens suitable in terms of day-age [16].

In recent years, researchers have developed a variety of digital image processing and pattern recognition techniques that use camera traps for objection detection and classification, which identify species accurately and concisely in agriculture [17,18,19,20,21,22,23]. In Ref. [2], Ren et al. improved the accuracy of chicken day-age detection. They proposed an attention encoder structure to extract chicken image features, trying to improve the detection accuracy. To cope with the imbalance of the dataset, various data enhancement schemes such as Cutout, CutMix and MixUp were proposed to verify the effectiveness of the proposed attention encoder. By applying the attention encoder structure, they can improve the accuracy of chicken age detection to 95.2% and they also designed a complete image acquisition system for chicken houses and a detection application configured for mobile. However, when the number of captured cameras proliferates, the strategy based on single-point training will lead to long training time and how to utilize the large amount of edge computing power will be the key to solving this problem.

These studies provide the basis for research on classification of chickens based on computer vision and point the way forward. Based on the above discussion, this paper proposes a high-precision federated learning-based chicken 100-day-old classification model that can be applied to edge computing scenarios. The main contributions of this paper are as follows:

1. This paper proposes a dual-ended adaptive federal learning framework that can be adapted to clients with different computing powers.
2. In order to adapt to edge computing scenarios, the mainstream classification models have been lightened to run on low computing power platforms.
3. This paper conducted extensive experiments to validate and analyze the robustness of the proposed method and finally achieved 95.3% accuracy on our dataset.

## 2. Related Work

**Figure 1:**
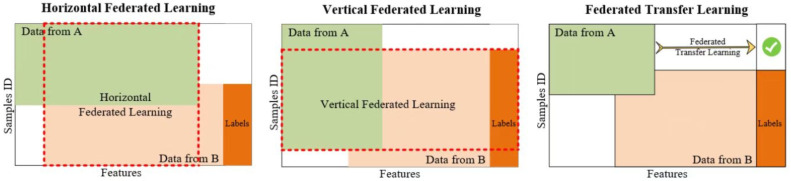
Illustration of three types of federated learning: horizontal federated learning, vertical federated learning and federated transfer learning.

Federated learning was first proposed by Google in 2016 [24] and was originally used to solve the problem of updating models locally for Android phone end users. It is essentially a distributed machine learning technique or machine learning framework. The goal of federated learning is to enable co-modeling and improve the effectiveness of AI models while ensuring data privacy and security and legal compliance. Each entity involved in joint modeling is called a participant and based on the distribution of data across multiple participants, joint learning is divided into three categories: horizontal vertical joint learning, joint transfer learning and joint learning, as shown in Figure 1.

### 2.1. Horizontal Federal Learning

The essence of cross-sectional federation learning is the union of samples, which apply to the scenario when the participants have the same business model but reach different customers, i.e., more overlapping features and less overlapping users, e.g., banks in different regions have a similar business (similar features) but different users (different samples). The learning process is shown below:

1. The participants each download the latest model from server A.
2. Each participant trains the model using local data and the encrypted gradients are uploaded to Server A, which aggregates the gradients of each user to update the model parameters.
3. Server A returns the updated model to each participant.
4. Each participant updates his model.

In traditional machine learning modeling, the data needed to train a model is usually collected in a data center prior to training the model and making further predictions. Horizontal composite learning is a sample-based distributed model training where all the data for this training are distributed across different computers. Each computer downloads the model from the server, then trains the model based on the local data and sends any parameters that need to be updated back to the server. Based on the parameters returned by the different computers, the server compiles and updates the model and then sends the latest model to the different computers.

In this process, there is no communication and dependency between machines, each machine can also predict independently when predicting and the models under each machine are identical and complete, which can be said to be sample-based distributed model training. Google first uses horizontal federation to solve the model local update problem for Android mobile end users.

### 2.2. Vertical Federal Learning

The essence of vertical federation learning is the union of features, which is suitable for scenarios with more overlapping users and less overlapping features, such as a superstore and a bank in the same area, where they reach users who are both residents of the area (same sample) but have different businesses (different features). In the traditional machine learning modeling process, two parts of data need to be pooled into one data center and then the features of each user are joined into one piece of data for training the model, so both sides must have user intersection and one side to have a label. The first step is to encrypt the sample alignment. This is done at the system level so that non-intersecting users are not exposed at the enterprise perception level; the second step is to align the samples for model encryption training, as shown below:

1. The public key sent by the third party C to A and B, which is used to encrypt the data to be transmitted.
2. A and B compute the intermediate results of the features associated with themselves, respectively, and encrypt the interactions, which are used to derive the respective gradients and losses.
3. A and B compute their respective encrypted gradients and add masks to send to C, while B computes the encrypted losses to send to C.
4. C decrypts the gradient and loss and passes it back to A and B. A and B remove the mask and update the model.

### 2.3. Federated Transfer Learning

**Figure 2:**
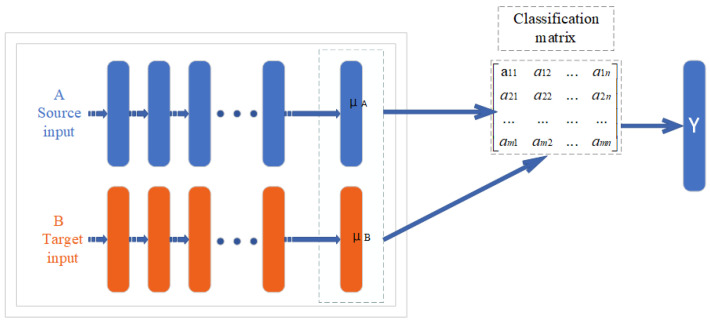
The process of federation migration.

Federated transfer learning can be considered when there is little feature and sample overlap among participants, such as the federation between banks and supermarkets in different regions. It is mainly applicable to scenarios where deep neural networks are the base models. The steps of federated transfer learning are similar to those of longitudinal federated learning, except that the intermediate transfer results are different (in fact, the intermediate transfer results are different for each model). The process of federation migration is shown in Figure 2.

Federated learning is reflected in the fact that A and B can learn a model together by securely interacting with intermediate results and transfer learning is reflected in the fact that B migrates the classification ability of A.

## 3. Materials and Methods

### 3.1. Dataset Analysis

The dataset used in this study was approved by the Animal Ethics Committee of China Agricultural University and was collected from a private rearing company’s chicken coop in Guangdong Province from the first day of life to the 100th day of life, using smartphones, Canon cameras and CCTV cameras. The distribution of the dataset at different days is shown in Table 2.

**Figure 3:**
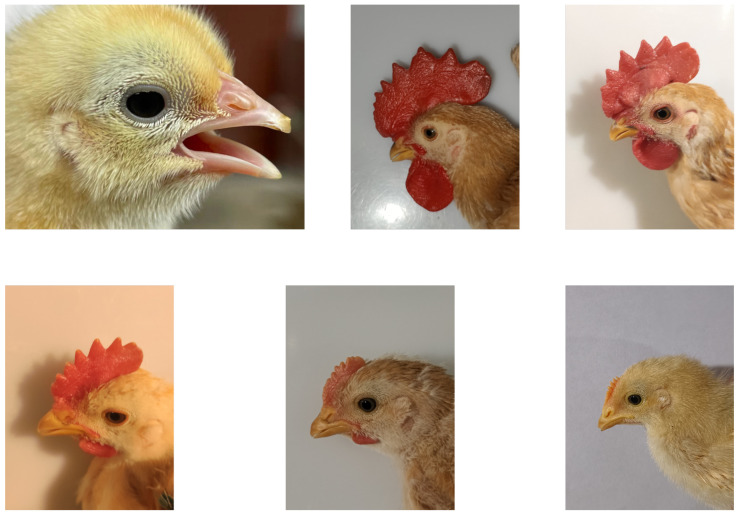
Exhibition of the chicken dataset from different day-ages.

The Guangdong Academy of Agricultural Sciences, China, manually collected the dataset adopted in this paper. Researchers used a Canon 5D digital camera to capture these images from January 2021 to October 2022. Figure 3 displays details of this dataset and each image’s resolution is 6720×4480.

From the above table, it can be seen that the dataset used in this paper has the following characteristics:

1. Our dataset is huge and it is time consuming and difficult to train and learn on a single client.
2. Our dataset has many classes, for a single classification model, it is difficult to classify accurately.
3. Our dataset is unevenly distributed, with some having a large number of day-old images and others having a small number of images or even missing ones.

Due to the above characteristics of the dataset and the usage scenario, it is difficult to train a single model with a single client and get a high accuracy model, so this paper proposes a distributed training method based on federal learning.

### 3.2. Dataset Rebalancing and Recovery

As stated in Section 3.1, the dataset in this paper is not balanced. In order to prevent the categories with few images from being ignored by the model, this paper first targets the weak categories in the dataset for rebalancing before training. The following three data enhancement methods are used:

1. Geometric transformations. Geometric transformations are geometric transformations of the image, including flipping, rotating, cropping, deforming, scaling and other operations.
2. Color transformations. The above geometric transformations do not change the content of the image itself; it may select a part of the image or redistribute the pixels. If you want to change the content of the image itself, it belongs to the color transformation class of data enhancement, including noise, blur, color transformation, erase, fill, etc.
3. Multi-sample data enhancement. Unlike single-sample data enhancement, multi-sample data enhancement methods use multiple samples to generate new samples, such as SMOTE [25] and SamplePairing [26].

For training, this paper follows the method proposed by [27], dividing all images into training and test sets in the ratio of 7:3 and the results mentioned in Section 4 are obtained in the test set.

### 3.3. Proposed Methods

**Figure 4:**
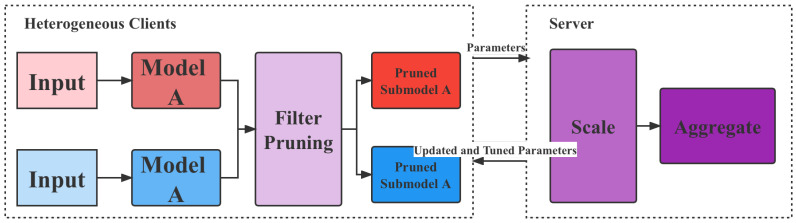
The flowchart of proposed method.

The proposed model flow is shown in Figure 4, which consists of three parts: client-side based CNN model, network lightweight transformation and bipartite feasible learning framework.

As shown in the figure above, this paper designs a lightweight network model for heterogeneous devices so that it can run on devices with poor computing power. Then, this paper trains it through a federation learning mechanism, updates its parameters and synchronizes it with all terminals.

#### 3.3.1. Dual-Ended Personalized Federal Learning Framework

The dual-ended personalized federation learning framework means that a resource-aware and data-directed model pruning component on each client uses local data to learn the filter importance of the initialized global model. It then performs filter pruning on the global model based on the learned filter importance and the client’s system resource budget. Since the learned filter importance integrates data distribution information, the pruned sub-model can be adapted to each client’s data distribution and resource capacity. After that, each client trains the pruned submodels in parallel and uploads the parameters of the submodels to the server for aggregation.

To efficiently aggregate the parameters of the submodels on the server, the scaling-based model aggregation component first scales the parameters according to the pruning rates of the submodels. Then, it performs a weighted average of the overlapping parameters of the submodels to update the global model. Before distributing the updated submodel parameters to the clients, the server-assisted model tuning component uses the global view of the server to adjust and further optimize the submodel structure. Specifically, it first calculates the similarity of the data distribution among the clients using the filter importance learned by each client. Based on the similarity information, it adjusts the retained filter indices of each submodel to follow the The proposed principle of individualization.

**Figure 5:**
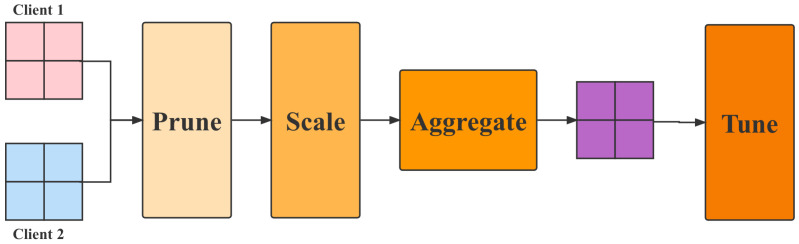
The flowchart of dual-ended personalized federal learning.

Finally, in the updated global model, the corresponding parameters of the adjusted filter are reduced and returned to each client for the next round of model training and communication. The workflow is shown in Figure 5.

#### 3.3.2. Classification Convolutional Neural Networks

##### AlexNet

AlexNet was proposed by Geoffrey and his student Alex and won the first place in the 2012 ILSVRC competition [27]. AlexNet has an eight-layer structure, with the first five layers being convolutional and the last three being fully connected. The AlexNet has the following characteristics:

1. AlexNet selects a nonlinear non-saturated ReLu function in the activation function. In terms of gradient decay speed in the training phase, the ReLu function is much faster than the nonlinear saturated functions, such as sigmoid function and tanh function, which are selected by traditional neural networks.
2. AlexNet runs on dual GPUs, each gpu is responsible for half of the network operations.
3. It uses local response normalization (LRN). For the non-saturated function, ReLU, there is no need to normalize its input, but Alex et al. found that adding LRN to the ReLu layer creates some form of lateral suppression, which improves the generalization ability of the network.
4. The pooling approach uses overlapping pooling. That is, the size of the pooling window is larger than the step size, making each pooling have an overlapping part. This overlapping pooling method has better results than the traditional non-overlapping pooling method and can avoid the overfitting phenomenon.

##### VGGNet

In 2014, Simonyan K. et al., proposed a new deep convolutional neural network: VGGNet [28]. The model has the following features: (1) the larger convolutional layers are replaced by convolutional layers composed of multiple small convolutional kernels, which on the one hand reduces the parameters and on the other hand is equivalent to performing more non-linear mapping, which can increase the model fitting ability; (2) the use of small pooling kernels allows the model to obtain more convolutional kernels making the number of channels of the feature map more numerous and the feature extraction more comprehensive; (3) instead of using fully connected layers, three convolutional layers are replaced, which makes the network no longer limited to fixed size inputs and can accept arbitrary size inputs.

The study points out that the VGGNet model has insufficient network depth to be explored and does not explore the effect of convolutional kernel width on network performance, while the network has too many parameters, reaching over 130 million parameters.

##### GoogLeNet

GoogLeNet is a deep neural network model based on the Inception module introduced by Google [29], which won the ImageNet competition in 2014. After increasing the depth and width of the network, the traditional deep learning model will encounter the following three problems: first, the more layers there are in the network, the easier the gradient disappearance problem is and the more difficult it is to optimize the model; second, with too many parameters, it is easy to produce overfitting; third, the larger the network and the more parameters, the greater the computational complexity and the more difficult it is to apply the model.

In this situation, GoogLeNet has proposed the method of inception, which is to take multiple convolutional or pooling operations and assemble them together into a network module and design the neural network in terms of modules to assemble the entire network structure. This has two advantages: firstly, it reduces the number of parameters significantly; secondly, this also improves the expressiveness of the network.

##### ResNet

**Figure 6:**
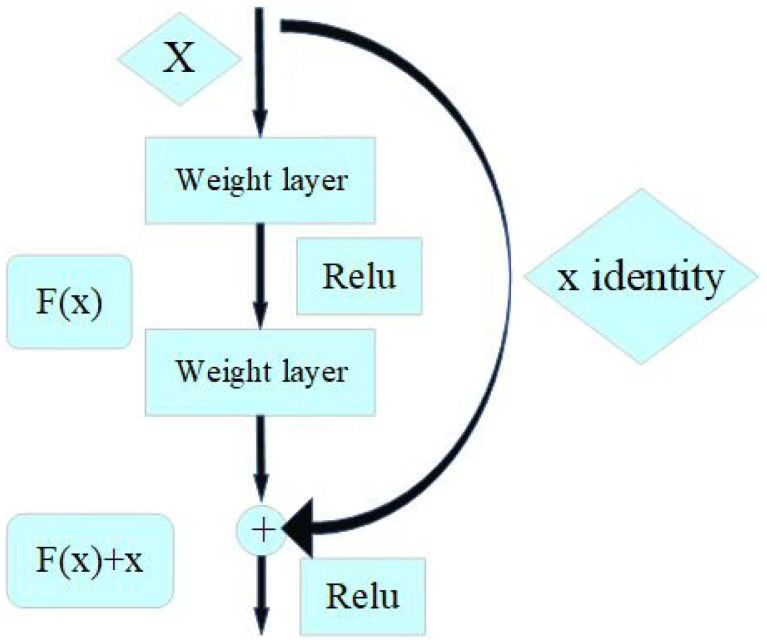
Shortcut connection module. ReLu is the activation function; F(x)+x is the original learning feature; *x* is the model input; F(x) is the residual.

There was a time when researchers had a common belief that as the number of layers of a model network increased, the more accurate the model was. However, as a result of the gradient explosion and gradient disappearance caused by the deepening of the network, the accuracy of the model decreases dramatically and without warning as the number of layers increases, a phenomenon known as degradation. To address these issues, He et al. propose a shortcut connection branching module that seeks a balance between linear and non-linear conversions [30], as shown in Figure 6.

When F(x)+x equals 0, the layer is only doing constant mapping at this point and the network performance does not degrade. In fact, the residuals will not be 0, which will also allow the stacking layer to learn new features on top of the input features and thus have better performance.

ResNet provides a solution to the problem of decreasing accuracy due to too many layers in the network, which greatly eliminates the problem of difficulty in training neural networks with too much depth.

#### 3.3.3. Network Lightweight Transformation

**Figure 7:**
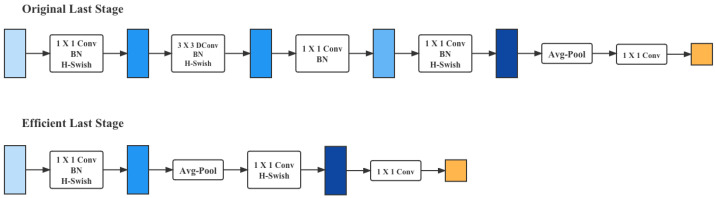
Redesign for layers with higher latency.

In order to enable the model to be inferred and trained in edge computing scenarios, this paper has modified the above network to be lightweight, using the following techniques:

1. Depthwise Separable Convolution. Depthwise separable convolution is a decomposition of the standard convolution into a depth convolution and a 1×1 point convolution. The depth convolution applies a single filter to each input channel and the point convolution combines the weighted output of the depth convolution. The effect of this decomposition is to greatly reduce the computational effort and model size. For example, given feature maps of $D_{f}\times D_{f}\times M$ generating feature maps of $D_{f}\times D_{f}\times N$, assume that the convolution kernel size is $D_{k}$. The ratio of the computational effort of the deep separable convolution to the standard convolution is $\frac{1}{N}+\frac{1}{D_{k}^{2}}$. Therefore, when using 3×3 convolution, this convolutional approach can reduce the computation by 8–9 times with little reduction in accuracy.
  In addition, for unstructured sparse matrices, the computational effort is less than that of dense matrices, but dense matrices are faster to compute because they are optimized using universal matrix multiplication at the bottom. Here the 1×1 convolution in the deep separable convolution does not need to be reordered and the matrix operation can be used directly. As for the deep convolution part, the number of parameters and the computation are very small and the optimization is calculated according to the normal convolution. Therefore, the depth-separable convolution is computed very quickly.
2. Linear Bottlenecks. The ReLu activation function in CNN corrupts the data in the low-dimensional space, while the high-dimensional space is less affected. Therefore, linear activation is used instead of ReLu in low-dimensional space and it has been experimentally shown that using linear layer in low-dimensional space is quite useful because it avoids too much information corruption by nonlinearity. In addition, if the output is a non-zero space in the form of a stream, using ReLu is equivalent to doing a linear transformation, which will not achieve the spatial mapping, so this paper uses a nonlinear activation in the non-zero space.
3. Inverted Residuals. Unlike ResNet, where the bottleneck residuals are hourglass-shaped, i.e., downscaled when passing through the 1×1 convolutional layer, this paper uses the spindle-shaped sort, which is upscaled at the 1×1 convolutional layer. This is because this paper uses a deeply separable convolution, the number of parameters is already extremely small and the generalization ability will be insufficient if this paper uses dimensionality reduction. The result is shown in Table 3.
4. Redesign for layers with higher latency. To reduce the latency and keep the high-dimensional spatial features, this paper moved some layers behind the average pooling layer, where the final feature set now only needs to be computed at a resolution of 1×1 instead of the original 7×7. The result of this design choice is that the computation of features becomes almost free in terms of computation and latency, as shown in Figure 7.
  Once the cost of this feature generation layer has been reduced, the previous bottleneck projection layer is no longer needed to reduce the computation. This observation allows one to remove the projection and filtering layers from the previous bottleneck layer, thus further reducing the computational complexity. The original and optimized phases are shown in the figure above.

### 3.4. Experiment Implementation

#### 3.4.1. Experimental Platform

This paper implemented the proposed method based on PyTorch and composed a mobile system with five heterogeneous devices and a host computer equipped with an NVIDIA Geforce RTX 3080 graphics card. These heterogeneous devices are common mobile platforms, including smartphones and development boards and are configured as shown in Table 4.

As shown in the table above, these devices have different processors, DRAMs and network environments. In addition, this paper provides a simple API for model tuning and supports the CNN models used in this paper, including AlexNet, VGG, GoogLeNet and ResNet, as mentioned in Section 3.3.2.

#### 3.4.2. Evaluation Metric

**Figure 8:**
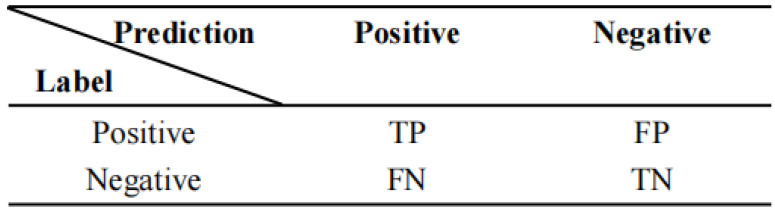
Matrix of classification metrics.

This paper uses the following three evaluation metrics: accuracy, precision and recall. First, this paper introduces the confusion matrix. A confusion matrix is a table often used in data science and machine learning to summarize the prediction results of a classification model, represented by a matrix with n rows and n columns, which summarizes the records in a dataset according to two criteria: the true category and the predicted category. As an example, the confusion matrix for the dichotomous classification task is shown in Figure 8.

*TP* denotes the number of samples that are positive and predicted to be positive, *FP* denotes the number of samples that are negative and predicted to be positive, *FN* denotes the number of samples that are positive and predicted to be negative and *TN* denotes the number of samples that are negative and predicted to be negative.

Based on the above discussion, the formula for accuracy is given by:

(1) $$Accuracy=\frac{TP+TN}{TP+TN+FP+FN}$$

Accuracy is the simplest and most intuitive evaluation metric in classification problems, but there are obvious drawbacks. For example, if 99% of the samples are positive, then the classifier only needs to predict positive all the time to obtain 99% accuracy, but its actual performance is very low. That is, when the proportion of samples from different categories is very unbalanced, the category with a large proportion tends to be the most important factor affecting the accuracy.

(2) $$Precision=\frac{TP}{TP+FP}$$

Both of these metrics are concerned with the proportion of samples that are correctly predicted by the model in the model prediction or the total. The recall is the proportion of actual positive samples that are predicted to be positive out of the actual positive samples and is calculated as:

(3) $$Recall=\frac{TP}{TP+FN}$$

The recall is intuitively the ability of a classifier to find all positive samples. The best value of recall is 1 and the worst value is 0.

## 4. Results and Discussion

### 4.1. Comparison of Classification Accuracy

This paper first compares the classification accuracy of a single classification network with that of our method based on ResNet, VGG and GoogLeNet. The experimental results are shown in Table 5.

It should be noted that our values in the above table are obtained by using the federal learning framework, while the other methods’ are of a single model. Therefore, it is understandable that there is such a huge difference in performance, but of course, a fairer comparison is developed later in this paper. From the results in the table, it can be seen that our method has a huge advantage over the single model in terms of accuracy and our method is 14.4 percentage points ahead of AlexNet in the accuracy metric. This fully demonstrates the effectiveness of our method.

### 4.2. Comparison of Lightweighted Network Accuracy

In this section, this paper compares the accuracy of the lightened network with the original network model, as shown in Table 6.

From the above table, the following conclusions can be drawn: (1) the light weighting of the model causes different degrees of performance degradation, among which the accuracy degradation of ResNet is the most obvious, probably due to the replacement of its residual structure; (2) however, the accuracy loss caused by light weighting is compensated when the federal learning framework is applied, in which the light weighted network combined with federal learning is compared with the original network in ResNet. In ResNet, the light-weighted network with federation learning achieves the same accuracy as the original network. This result shows that when using the federal learning framework, both model accuracy and model inference speed can be gained by using a lightweight network.

#### Base Model Replacement

In this subsection, this paper investigates the effect of model component replacement, the results are shown in Table 7.

As can be seen from the above table, federal learning works best when as many different submodels as possible are used. In the single model scenario, ResNet and GoogLeNet perform significantly better than VGG and Alexnet. Moreover, the data in rows 6–8 of the table show that ResNet is more important among all models, probably because it is more different from the other models and the performance loss of federated learning is the greatest when it is discarded. This result can be used to guide us in the selection of sub-models.

### 4.3. Resource Efficiency of Proposed Method

In our evaluation, outstanding resource reductions in terms of memory, computation and communication overheads are achieved compared to the baseline. The resource efficiency of the model in this paper can be further improved by optimizing its implementation. For example, compression techniques can be applied to the transmitted packets to further reduce the communication overhead. In addition, in the model of this paper, this paper performs resource analysis by theoretically calculating the resource requirements of the pruned submodel and accordingly determining the pruning rate of each device to meet its resource constraints. The details are shown in Table 8.

### 4.4. Robustness Analysis of Proposed Method

#### 4.4.1. Robustness with Different Number of Clients

Since the final learning effect of federated learning is determined by the integration of many clients, this subsection investigates the variation of the final effect of the model when the number of clients varies, as shown in Table 9.

First of all, the results in the above table are obtained when the dataset is divided equally. It can be seen that the accuracy of our model is lowest when there is only one device, which is expected considering that this paper only uses a lightweight model at this point. As the number of terminals increases, the accuracy of all models improves. Although some models show slight jitters in accuracy, such as AlexNet and GoogLeNet, the fluctuations are not significant. When the number of terminals reaches 4, the accuracy of this method is almost the same as that of the baseline; when the number of terminals reaches 5, the accuracy of this method reaches 96.1%, which exceeds all other models. This result shows that the proposed federal learning framework can improve the model performance more effectively when the number of terminals increases.

#### 4.4.2. Robustness under Different Data Segmentation

The performance of federal learning fluctuates when the datasets are distributed differently, especially when the datasets do not satisfy the condition of independent homogeneous distribution. Therefore, this subsection investigate the robustness of the model under different data partitions. The experimental results are shown in Table 10 by varying the client’s dataset distribution.

The experimental devices used are those mentioned in Table 4, including two Jetson Nana, two Huawei Mate 20 and one Samsung Smart Phone. The data distribution is randomly assigned to these edge computing devices, according to the scale in the table. From the above table, it can be seen that our method outperforms other baseline models regardless of the dataset segmentation approach. At the same time, almost all models perform better when equal partitioning is adopted, while all models are affected to different degrees when the models are unevenly distributed, such as 1:2:3:4 and AlexNet is the least robust in this respect.

### 4.5. Convergence Discussion

Section 4 and Section 4.4 presented extensive experiments to demonstrate that our model can converge on the dataset with different base models and a different number of clients.

In particular, Section 4.4.2 also experimented with different divisions of the dataset, although this paper evaluated the performance of our method by manual division and proved its convergence under different data division methods and settings.

### 4.6. Deploy Applications

**Figure 9:**
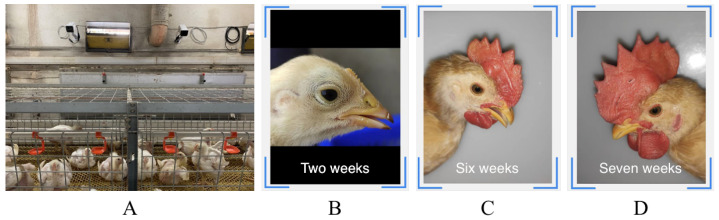
Application scenarios based on the methods in this paper. (**A**) is the deployment site; (**B**–**D**) are screenshots of the application.

By deploying the proposed method in a chicken farm edge computing device, as shown in Figure 9A, this paper designed a wechat SDK-based application that classifies the most appropriate edge devices and returns the classification results to the user device, as shown in Figure 9B–D.

## 5. Conclusions

This paper proposes a method based on a lightweight network and a federal learning framework to address the problem of low accuracy of chicken day-age recognition in practical scenarios. The main contributions of this paper are as follows:

1. This paper proposes a federated learning mechanism for heterogeneous terminals, so that model training and parameter updating can be efficiently implemented in different terminals.
2. This paper designs a lightweight model based on the mainstream CNN network and deploys it on the edge devices to address the general lack of computing power of terminal computing devices.
3. In this paper, a large number of experiments are conducted to verify the effectiveness of this approach. For accuracy, this method outperforms the baseline models by 14.4% to 96.1%; for latency, this model can reduce the latency by 90.1% with the same network environment.
4. Based on the wechat SDK, this paper develops the model for mobile applications and deploys it in real-world scenarios.

The method proposed in this paper has been validated in the scenario of chicken day-old detection. In future work, we will further extend this approach to other agricultural scenarios, especially to large-scale smart agriculture scenarios, such as animal detection and individual identification in large-scale livestock farming.

## Figures and Tables

**Table 1:** Information about chickens in diverse classification standards.

Phase Type	0–42	43–98	98–126
Two-part form	Chicks	Rearing chicken	Rearing chicken
Three-part form	Prophase of the chickling period	Metaphase of the chickling period	Anaphase of the chickling period

**Table 2:** Distribution of datasets between classes.

Day-Age	Images	Day-Age	Images	Day-Age	Images	Day-Age	Images
1	286	2	268	3	249	4	273
5	251	6	275	7	269	8	263
9	278	10	266	11	266	12	250
13	260	14	3176	15	3164	16	3150
17	3171	18	3140	19	3133	20	3140
21	3120	22	3117	23	3080	24	3050
25	3070	26	3066	27	3052	28	3040
29	3050	30	3024	31	2928	32	-
33	-	34	-	35	2916	36	2903
37	2933	38	2929	39	2932	40	2921
41	2922	42	2966	43	2928	44	2923
45	2961	46	2926	47	2925	48	2913
49	2922	50	3020	51	-	52	-
53	-	54	-	55	-	56	-
57	-	58	-	59	-	60	-
61	-	62	-	63	-	64	-
65	-	66	-	67	-	68	765
69	741	70	630	71	622	72	619
73	646	74	629	75	628	76	-
77	612	78	648	79	723	80	612
81	635	82	648	83	644	84	639
85	644	86	648	87	647	88	675
89	633	90	649	91	-	92	648
93	647	94	638	95	622	96	652
97	653	98	650	99	641	100	652

**Table 3:** Inverted residuals block transforming from *k* to $k^{\prime }$.

Input	Output	Operator
h×w×k	h×w×tk	1×1 conv2d
h×w×tk	$\frac{h}{s}\times \frac{w}{s}tk$	3×3
$\frac{h}{s}\times \frac{w}{s}\times tk$	$\frac{h}{s}\times \frac{w}{s}k^{\prime }$	linear 1×1 conv2d

**Table 4:** Summary of heterogeneous devices.

Device	Number	Processor	Network	DRAM
NVIDIA Jetson Nano	2	Cortex A57	Ethernet	8 GB
HUAWEI Mate 20	2	Kirin 980	Wifi	6 GB
Samsung Galaxy Fold 2	1	Snapdragon 865	Wifi	8 GB

**Table 5:** Comparison of single CNN and our method.

Model	Accuracy	Recall	Precision
AlexNet	81.7%	78.1%	81.6%
VGG	85.2%	81.7%	85.5%
GoogLeNet	90.1%	87.6%	89.4%
ResNet	90.3%	87.8%	91.0%
Ours (based on 3 models)	96.1%	92.9%	95.8%

**Table 6:** Accuracy of original models and lightweighted models without federal learning (FL).

Model	Origin	Lightweight	Origin with FL	Lightweight with FL
AlexNet	81.7	80.5	85.2	84.8
VGG	85.2	82.6	89.1	88.4
GoogLeNet	90.1	86.9	93.7	93.3
ResNet	90.3	84.2	93.8	93.8

**Table 7:** Accuracy of different submodels.

AlexNet	VGG	GoogLeNet	ResNet	Accuracy
✓	✓	✓	✓	96.1
✓	-	-	-	85.2
-	✓	-	-	89.1
-	-	✓	-	93.7
-	-	-	✓	93.8
-	✓	-	✓	93.3
-	-	✓	✓	93.5
✓	-	✓	-	92.6

**Table 8:** Lightweighted models’ overhead versus original models’.

	Original	Lightweighted
Inference Latency	3.1 ms	0.7 ms
Transform Latency	27.5 ms	11.2 ms

**Table 9:** Accuracy of different models with different client numbers.

Numbers	AlexNet	VGG	GoogLeNet	ResNet	Ours (Based on 3 Models)
1	81.7	85.2	90.1	90.3	86.9
2	83.5	87.1	93.3	92.8	88.2
3	85.8	88.4	93.5	93.8	90.8
4	85.7	88.7	93.8	94.1	93.1
5	85.2	89.1	93.7	93.8	96.1

**Table 10:** Accuracy of different models with different dataset distribution.

Distribution	AlexNet	VGG	GoogLeNet	ResNet	Ours (Based on 3 Models)
1:1:1:1:1	85.2	89.1	93.7	93.8	96.1
1:2:3:4	83.5	88.2	92.1	93.6	95.3
1:3:3:3	85.6	88.2	93.8	93.8	95.8

## Data Availability

The full datasets can be made available by the authors upon reasonable request.
